# Adjusting Some Properties of Poly(methacrylic acid) (Nano)Composite Hydrogels by Means of Silicon-Containing Inorganic Fillers

**DOI:** 10.3390/ijms231810320

**Published:** 2022-09-07

**Authors:** Claudia Mihaela Ninciuleanu, Raluca Ianchiș, Elvira Alexandrescu, Cătălin Ionuț Mihăescu, Sabina Burlacu, Bogdan Trică, Cristina Lavinia Nistor, Silviu Preda, Cristina Scomoroscenco, Cătălina Gîfu, Cristian Petcu, Mircea Teodorescu

**Affiliations:** 1 National Institute for Research and Development in Chemistry and Petrochemistry-ICECHIM, Spl. Independentei 202, 060021 Bucharest, Romania; 2 Department of Bioresources and Polymer Science, Faculty of Applied Chemistry and Materials Science, Politehnica University of Bucharest, 1-7 Gh. Polizu Street, 011061 Bucharest, Romania; 3 Institute of Physical Chemistry “Ilie Murgulescu”, Romanian Academy, Spl. Independentei 202, 6th District, P.O. Box 194, 060021 Bucharest, Romania

**Keywords:** nanocomposites, poly(methacrylic acid), hydrogel, montmorillonite, Laponite, pyrogenic silica, water decontamination

## Abstract

The present work aims to show how the main properties of poly(methacrylic acid) (PMAA) hydrogels can be engineered by means of several silicon-based fillers (Laponite XLS/XLG, montmorillonite (Mt), pyrogenic silica (PS)) employed at 10 wt% concentration based on MAA. Various techniques (FT-IR, XRD, TGA, SEM, TEM, DLS, rheological measurements, UV-VIS) were used to comparatively study the effect of these fillers, in correlation with their characteristics, upon the structure and swelling, viscoelastic, and water decontamination properties of (nano)composite hydrogels. The experiments demonstrated that the nanocomposite hydrogel morphology was dictated by the way the filler particles dispersed in water. The equilibrium swelling degree (SDe) depended on both the pH of the environment and the filler nature. At pH 1.2, a slight crosslinking effect of the fillers was evidenced, increasing in the order Mt < Laponite < PS. At pH > pKa_MAA_ (pH 5.4; 7.4; 9.5), the Laponite/Mt-containing hydrogels displayed a higher SDe as compared to the neat one, while at pH 7.4/9.5 the PS-filled hydrogels surprisingly displayed the highest SDe. Rheological measurements on as-prepared hydrogels showed that the filler addition improved the mechanical properties. After equilibrium swelling at pH 5.4, G’ and G” depended on the filler, the Laponite-reinforced hydrogels proving to be the strongest. The (nano)composite hydrogels synthesized displayed filler-dependent absorption properties of two cationic dyes used as model water pollutants, Laponite XLS-reinforced hydrogel demonstrating both the highest absorption rate and absorption capacity. Besides wastewater purification, the (nano)composite hydrogels described here may also find applications in the pharmaceutical field as devices for the controlled release of drugs.

## 1. Introduction

Composite materials have increasingly attracted the attention of researchers within the last few decades due to their properties being far superior to simple materials [1,2,3,4,5]. A special class of such materials is represented by composite hydrogels, which may generally be defined as hydrophilic polymer networks capable of retaining certain quantities of aqueous solutions in the presence of small particles acting as reinforcing agents [6,7,8]. Composite hydrogels have been and are being studied a great deal due to their improved mechanical, electrical, thermal, and optical properties, absorption capacity, and sensitivity to different stimuli as compared to their unreinforced counterparts [9,10]. The improved properties of the composite hydrogels are a consequence of the synergistic effect of the individual phases, i.e., the polymer matrix and the inorganic filler [11]. Various nano- and microparticles have been used as reinforcing agents for hydrogels over the years, such as graphene and its derivatives (graphene oxide, carbon nanotubes) [12,13], bioactive glass [14], metal nanoparticles (Ag, Au), and metal oxides (Fe_3_O_4_, Fe_2_O_3,_ alumina, etc.) [15], the distinct properties of the reinforcing agents allowing the design of the final hydrogel in agreement with the required characteristics.

A special class of fillers for hydrogels is represented by materials that contain Si-O groups within their structure. This category includes various types of silica-based nanoparticles [16], such as pyrogenic silica [17,18,19,20] and layered clays such as montmorillonite (Mt) [21] and Laponite (Lap) [22], which have the advantage of being cheaper than the agents listed above. Among them, clays have attracted more and more attention as fillers due to their remarkable properties, such as high specific surface area and adsorption capacity, optimal rheological properties, chemical inertia, and low toxicity, Lap and Mt being the most used for hydrogel reinforcement [6,21,22,23,24]. From a structural point of view, they have similarities, as both belong to the smectite class. Clays are usually micro- or nanometric particles made up of layered sheets of 2D silicate (Figure S1). The empirical formula of Mt is (Na,Ca)_0.33_(Al,Mg)_2_(Si_4_O_10_)(OH)_2_·nH_2_O [25]. Its layers, approximately 1 nm thick and 100 nm × 100 nm width × length, are composed of two tetrahedral sheets formed by O-Si-O bonds and an octahedral sheet formed by O-Al(Mg)–O bonds [26]. The Lap layers are instead in the form of disks of approximately 25 nm diameter and 0.92 nm thickness [26]. The empirical formula of Lap reported in the literature is Na_0.7_^+^[(Si_8_Mg_5.5_Li_0.3_)O_2_(OH)_4_]^−0.7^ [27]. Commercial Lap is available in different varieties: XLG, XLS, RD, RDS, XLG, and XLS possessing a higher purity and a lower content of heavy metals than Mt. In aqueous dispersions with concentrations higher than 2%, Laponite XLG can form the so-called “house of cards” structure, leading to gelation due to the ionic interactions among the positively-charged edges of some sheets and the negatively-charged faces of others [27]. Laponite XLS does not form the “house of cards” structure because it is modified with pyrophosphate ions [28,29].

Pyrogenic (fumed) silica nanoparticles (Figure S1) are obtained by SiCl_4_ pyrolysis at temperatures above 1000 °C and are in the form of silica spheres with 5–30 nm diameter, forming particle chains of 100–1000 nm length. These particle chains lead eventually to porous networks that extend up to 250 μm. The primary particles are composed of SiO_4_ tetrahedra, while on the surface they display both oxygen atoms belonging to siloxane groups (Si-O-Si) and silicon atoms from silanol groups (Si-OH) [30]. Pure pyrogenic silica is hydrophilic and has a high surface energy due to the presence of these groups [31]. Pyrogenic silica nanoparticles are stable in aqueous solutions [32], but unlike layered silicates, they do not dissociate with the formation of free ions [17].

Both clays [24,33] and pyrogenic silica nanoparticles [17,18,19] have proven their effect on hydrogels, especially by increasing their mechanical and/or thermal properties or changing the swelling degree, but silica nanoparticles are less studied as reinforcing agents for hydrogels as compared to clays. Incorporating clays into the hydrogel matrix is also a good way to increase the final absorption properties of the hydrogel because they can absorb positively-charged pollutants, such as heavy metals and cationic dyes [34]. A study in this regard was conducted by Peng et al., who synthesized a hydrogel from cellulose and Mt that was tested for the adsorption of methylene blue, obtaining an absorption capacity of over 90% [35]. For methylene blue absorption as well, Yi et al. synthesized hybrid hydrogels based on polyacrylamide, sodium humate, and Lap [36].

Acrylic hydrogels are used in many biomedical fields such as drug delivery, intraocular and contact lenses, bone cement for orthopedics, dressings, and implants for regenerative medicine [37]. Another possible application is for removing pollutants from wastewater through possible interactions between the polymer functional groups and the pollutant. It is well known that one of the big challenges of the environment is the pollution of water by dyes, and various studies have shown that hydrogels represent a promising way to solve this problem [38]. Among the acrylic hydrogels, those based on methacrylic acid (MAA) have an important share due to their remarkable properties, such as pH-sensitive character, mucoadhesive characteristics [33], good absorption properties [39], lack of toxicity, etc. [40]. Composite hydrogels based on poly(methacrylic acid) (PMAA) were obtained by reinforcing hydrogels with different types of clays: bentonite [41], Mt [42,43,44], modified Mt [11], Lap [45,46], kaolin [47], gold nanoparticles [48], pyrogenic silica [17], and carbon nanotubes [49]. By introducing different nanoparticles into the PMAA matrix, properties such as the mechanical and thermal ones, and water absorption have been improved. Potential applications for PMAA-based composite hydrogels reported in the literature include controlled drug release [48,50,51] or treatment of contaminated water [39,44,49]. Regarding the employment of PMAA hydrogels as dye absorbents, there are only two studies on this subject, as far as we know, describing the use of both unreinforced and zeolite-reinforced hydrogels in connection with Yellow 28 dye [39,52].

The present work aims to show how the main properties of the PMAA hydrogels can be engineered by means of several silicon-based fillers, namely Laponite XLS (XLS), Laponite XLG (XLG), montmorillonite (Mt), and two commercial brands of pyrogenic silica differing mainly by the particle size and zeta potential of their 1.5% aqueous dispersion. For this purpose, the effect of these fillers on the structure and swelling, viscoelastic properties, and water decontamination ability of the (nano)composite hydrogels was comparatively studied in correlation with the filler characteristics. The hydrogels obtained were structurally characterized by FT-IR spectroscopy, X-ray diffractometry, thermogravimetric analysis, and electron microscopy (SEM, TEM). The viscoelastic properties were studied for both as-prepared and after equilibrium swelling hydrogels, while their swelling capacity was analyzed in aqueous media with various pHs, as a function of the nature of the reinforcing agent. Finally, the absorption properties of two cationic dyes, namely methylene blue and crystal violet, which are often found in the wastewater from several industries, were studied. To the best of our knowledge, this is the first report of such a comparative study of the influence of various reinforcing nano-agents, in particular clays and pyrogenic silica, in correlation with their characteristics, on the properties of some pH-sensitive PMAA-crosslinked hydrogels. This study also comparatively shows, for the first time, how the cationic dye absorption ability of a composite hydrogel depends on the reinforcing agent used. It should also be mentioned that the nanocomposite PMAA hydrogels reinforced with pyrogenic silica have already been reported in a paper that analyzed only the physical interactions occurring in the nanocomposite hydrogel [17]. Unlike that paper, the present work provides, for the first time, the extended characterization of these hydrogels, both structurally and in terms of rheological and swelling properties. The results presented in this paper may be useful in selecting an appropriate filler in order to adjust the properties of a PMAA hydrogel in agreement with the intended application. Previously, Zhang and Wang [Zhang, J; Wang, A. Study on superabsorbent composites. IX: Synthesis, characterization and swelling behaviours of polyacrylamide/clay composites based on various clays. React. Func. Polym. 2007, 67, 737–745] compared the effect of five clays (attapulgite, kaolinite, mica, vermiculite and Na^+^-montmorillonite) upon the thermal stability and swelling properties of some superabsorbent polyacrylamide hydrogels.

## 2. Results and Discussion

The studied hydrogels were obtained by the radical copolymerization of MAA with N,N’-methylenebisacrylamide (BIS) in aqueous solution, in the presence of ammonium persulfate (APS) as the initiator, by using different reinforcing agents (XLG, XLS, and Mt clays and two types of pyrogenic silica nanoparticles—HDK and FS—differing mainly by the particle size and zeta potential of their 1.5% aqueous dispersion). The characteristics of both reinforcing agents in water and their aqueous dispersions, determined by us, are displayed in Table 1. According to the measured zeta potential (Table 1), the aqueous dispersions of these fillers are stable over time and allow the acquisition of hydrogels with a uniform distribution of the reinforcing agent. Measurement of their particle size in 1.5 wt% aqueous dispersion showed that only XLG and XLS were exfoliated and dispersed as individual sheets. Mt was in the form of particles composed of non-exfoliated stacked clay layers, possibly mixed with individual sheets, while FS and HDK appeared as agglomerations of silica nanospheres (Table 1). In addition, the measurement of the pH of the filler aqueous dispersion indicated the presence of ionized basic functional groups in the case of clays, while the Si-OH functional groups on the surface of the pyrogenic silica particles were shown to be practically non-ionized in DI (pH ≈ 5.4).

The control hydrogel was prepared from an aqueous solution containing 15 wt% MAA and 2 mol% BIS based on MAA, plus the initiator (APS, 1 mol% to MAA), whereas in the case of (nano)composite hydrogels, reinforcing agents in a proportion of 1.5 wt% to the whole reaction mass were added. Hydrogels are indicated by an “H” followed by the abbreviation for the reinforcing agent. For example, “H” indicates the control hydrogel without reinforcing agent, while “HXLG” and “HHDK” stand for the hydrogels having Laponite XLG and HDK N20 pyrogenic silica as reinforcing agents, respectively.

The hydrogels were structurally characterized, and their viscoelastic and swelling properties were also investigated. The viscoelastic properties were investigated both in as-prepared state, when the composition of the hydrogels was the same in all cases, as well as after swelling at equilibrium. In addition, for a correct interpretation of the results, the water absorption/swelling degree was determined after the purification of the hydrogels and calculated only in relation to the amount of polymer in the hydrogel, excluding the mass of reinforcing agent incorporated in the case of composite hydrogels (Equation (6)). Because both the viscoelastic properties of hydrogels and their swelling degree depend on monomer conversion, this was determined in each case (Equation (1)). The results showed very high conversions (92–95%), which allowed comparison of the rheological and swelling measurements in all cases.

### 2.1. Hydrogel Structure

The structure of the synthesized hydrogels was studied by FT-IR spectroscopy, X-ray diffraction (XRD), electron microscopy (SEM, TEM), and thermogravimetric analysis (TGA) measurements. FT-IR spectroscopy was used to identify the interactions between the polymer matrix and each reinforcing agent, and also to observe the influence of the pH of the swelling aqueous medium upon the hydrogel structure. By comparing the FT-IR spectra of the (nano)composite hydrogels swelled in deionized water (pH 5.4) with both the control hydrogel and corresponding reinforcing agent (Figure S2), a strong solid-state interaction between the polymeric matrix and the inorganic agent was revealed by the shift of the Si-O band, characteristic to both clays and pyrogenic silica, to higher wavenumber values. The Si-O band was present in the spectrum of xerogels as a shoulder more (HFS, HDK, Figure S2d,e) or less (HXLG, HXLS, Figure S2a,b) pronounced or as a well-defined peak (HMt, Figure S2c).

**Figure 1:**
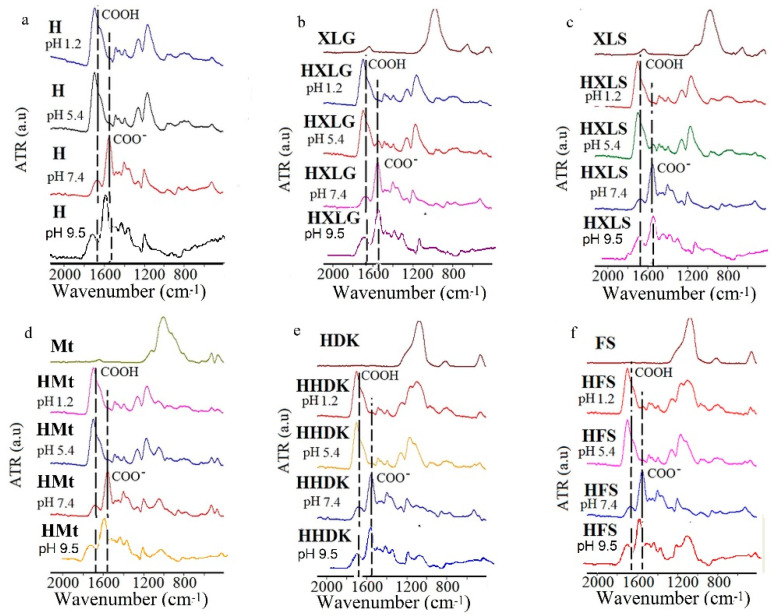
FT-IR spectra of (nano)composite hydrogels swelled at different pH values. (**a**) H; (**b**) HXLG; (**c**) HXLS; (**d**) HMt; (**e**) HHDK; (**f**) HFS.

Due to the pH-sensitive nature of PMAA hydrogels, their contact with aqueous solutions with different pHs causes changes of the contained functional groups, leading to different swelling behavior. To observe these changes, the hydrogels were analyzed by FT-IR after swelling at pH 1.2, 5.4, 7.4, and 9.5; drying; and grinding (Figure 1). The FT-IR spectra of the hydrogels swelled at pH 7.4 and 9.5 displayed a band characteristic of the carboxylate group at 1537 cm^−1^, simultaneously with the decrease of the COOH band from 1650 cm^−1^. This may be explained by the basic character of the swelling medium [53], whose pH was appreciably higher than the pKa value of the MAA units in the hydrogel. No notable differences were seen between the spectra at pH 7.4 and 9.5, although the hydrogels behaved differently at these pH values from a swelling point of view (see below). The decrease of pH to 1.2 led to the conversion of all hydrogel groups to COOH, and therefore, to the disappearance of the COO^−^ band in all spectra. At pH 5.4, which is the pH of the deionized water (DI) in which the hydrogels were synthesized and purified, different situations were encountered in the case of various nanocomposite hydrogels.

Thus, the FT-IR spectra of HXLG and HXLS (Figure 1b,c) displayed the COO^−^ group characteristic band, but of a much lower intensity than at pH 7.4/9.5, which can be explained by the basic groups contained by Lap clays, as proven by the basic pH of their aqueous dispersions (Table 1). They reacted with MAA in the hydrogel synthesis step to form COO^−^ groups, which were then preserved in the hydrogel structure after the purification step [45]. Very interesting is the fact that, although the aqueous dispersion of Mt also had a basic pH, practically identical to that of Lap, the FT-IR spectrum of HMt swelled to pH 5.4 did not show the COO^−^ characteristic band (Figure 1d). This may be explained by the smaller number of COO^−^ groups in the hydrogel formed in the synthesis stage as compared to the Lap-reinforced hydrogels. The smaller number of carboxylate groups was probably due to the much weaker exfoliated structure of Mt in water, leading to a lower contact with the monomer. The COO^−^ group band was not observed at pH 5.4 in the case of HHDK and HFS, due to the lack of basic character of the pyrogenic silica aqueous dispersions (Table 1). As it will be shown below, some of the COOH groups were ionized at pH 5.4 because of the lower pKa of the MAA units, but their concentration was probably too small to become visible in the FT-IR spectrum of H hydrogel (Figure 1a). The presence of COO^−^ groups in the hydrogels, as indicated by the FT-IR spectra, correlated very well with the swelling experiments that will be presented later in Section 2.2.

**Figure 2:**
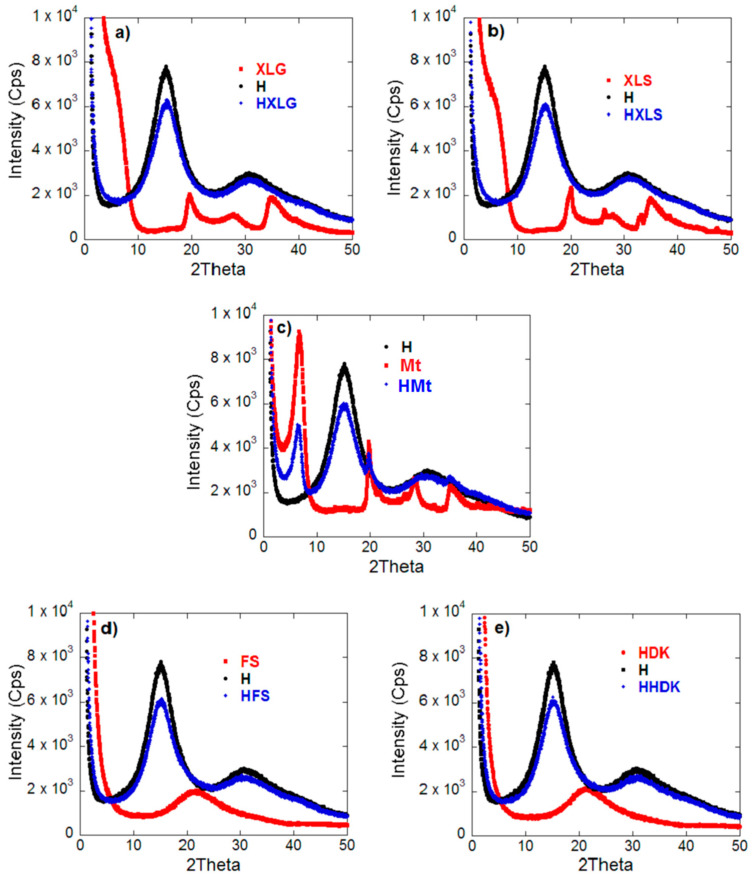
XRD spectra of both hydrogels investigated and reinforcing agents used. (**a**) HXLG; (**b**) HXLS; (**c**) HMt; (**d**) HFS; (**e**) HHDK.

The XRD analysis revealed the complete disappearance of the clay characteristic peaks in the case of Lap-reinforced hydrogels (Figure 2a,b), proving that exfoliated nanocomposite hydrogels were obtained [54]. In the case of Mt-reinforced xerogels (Figure 2c), the Mt characteristic reflection at 2θ = 6.6° was present, indicating the formation of intercalated composite hydrogels [42]. Pyrogenic silica-reinforced hydrogels were also characterized by XRD analysis (Figure 2d,e). The X-ray diffraction spectra of the silica nanoparticles showed only a wide band, indicative of the amorphous nature of commercial silica [55]. This band was no longer present in the corresponding hydrogel spectra, suggesting a strong polymer matrix-silica interaction.

**Figure 3:**
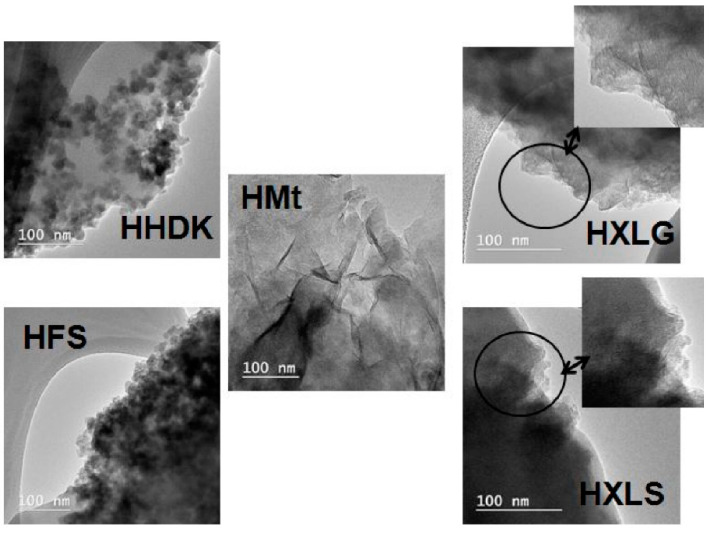
TEM images for hydrogels reinforced with various reinforcing agents.

The TEM analysis of HHDK and HFS xerogels showed that the fillers were dispersed within the xerogel in the form of approximately spherical particles of several tens of nanometers diameter, associated in large groups (Figure 3). In the case of the Mt-reinforced xerogel, the multi-layer clay particles were visible in the polymer matrix, suggesting the formation of intercalated nanocomposites, while for the XLS- and XLG-filled xerogels the TEM micrographs proved the exfoliation of the clay, as indicated by the single Lap layers with random orientation (Figure 3). Therefore, the TEM analysis supported the conclusions obtained by the XRD measurements. It should also be noted that the filler particles preserved in the hydrogel the structure they had in aqueous dispersion, i.e., isolated sheets in the case of XLG and XLS, stacked clay layers for Mt, and agglomerated spherical nanoparticles in the case of FS and HDK, as revealed by the DLS measurements.

The SEM analysis of the hydrogels swollen in DI and lyophilized revealed a structure with large open and interconnected pores in the case of both unreinforced and reinforced hydrogels (Figure S3).

**Figure 4:**
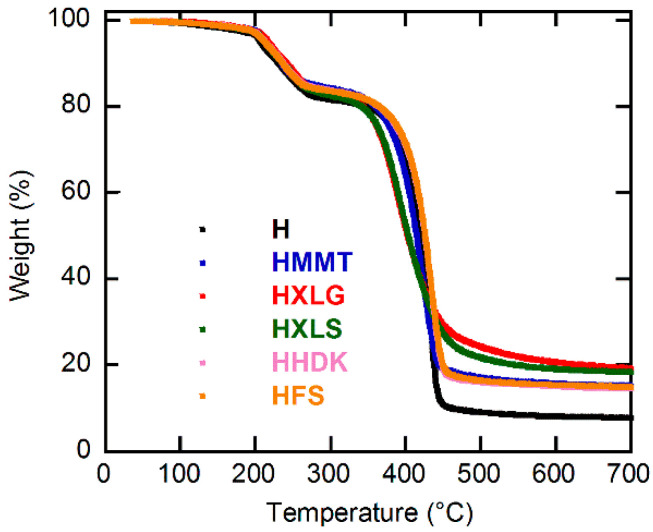
TGA curves for the composite xerogels obtained.

The TGA investigation of the obtained (nano)composite xerogels showed an increased residue amount at 700 °C as compared with the unreinforced one (Figure 4, Figure S4, and Figure S5, and Table 2), thus proving once again the presence of the inorganic filler within the polymer matrix. The thermogravimetric curves (Figure 4, Figure S3, and Figure S4) showed three decomposition steps. The first step (0–120 °C) was characteristic for the evaporation of the moisture existing in the xerogel and the second step (120–300 °C) was ascribed to both polymer dehydration by inter- and intramolecular water removal and decarboxylation of COOH groups, while the total decomposition of the PMAA chains took place in the third stage (300–700 °C) [50]. The inclusion of the reinforcing agents into the PMAA matrix did not lead to an appreciable change of the decomposition temperature of the polymer. However, some small differences could still be observed regarding the third stage, which seemed to start earlier for Lap samples, followed by the Mt sample and then the pyrogenic silica samples. This may be explained by the basic character of both Laponite and Mt (Table 1), which promoted the decomposition of PMAA chains, unlike the pyrogenic silica samples. As Lap was better dispersed within the xerogels than Mt, its effect on the polymer decomposition was more pronounced than for Mt, and therefore the PMAA chains started to decompose at a lower temperature in the case of Lap-filled samples. One should mention that, according to literature data, the presence of the inorganic component into organic–inorganic nanocomposites does not necessarily increase the thermal resistance of the material, being reported both increase [56] and decrease [57] of the thermal stability of the material, depending on its composition.

### 2.2. Hydrogel Swelling

**Figure 5:**
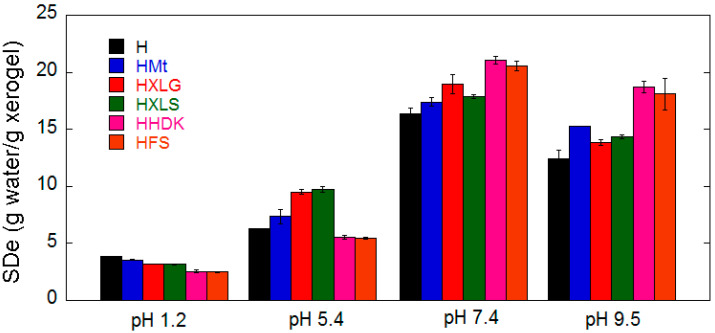
Equilibrium swelling degree in four different pH media.

The investigation of the swelling properties of the PMAA composite hydrogels at four different pHs (1.2, 5.4, 7.4, 9.5) at 25 °C showed a different behavior of the hydrogels, depending on both pH and reinforcing agent. Due to the pH-sensitive nature of PMAA-based hydrogels, the equilibrium swelling degree increased with increasing pH from 1.2 to 5.4 and further to 7.4/9.5, regardless of the nature of the filler, as expected, but at constant pH, hydrogels with different reinforcing agents behaved differently (Figure 5).

In a strong acidic environment (pH 1.2), the reinforced hydrogels swelled less than H, most likely because of the slight crosslinking effect of the fillers [44]. The additional crosslinking effect induced by the filler could be best identified at this pH because no COO^−^ groups formed due to the basicity of the medium or the reinforcing agent being present (Figure 1) [43,45,58]. An estimation of the structural parameters of the hydrogels at this pH (Equations (2)–(5)) showed an increase in the crosslinking density ($\rho_{c}$) as a function of the filler (H < HMt < HXLG ≈ HXLS < HHDK ≈ HFS, Table 3), probably determined by the number of additional PMAA network-reinforcing agent interactions.

The pH increase to 5.4 (deionized water) led to a general SDe increase and also to a different swelling behavior of hydrogels as a function of the reinforcing agent in comparison with their behavior at pH 1.2 (Figure 5). The general SDe increase was due to the ionization of a part of the COOH groups (pKa_MAA_ ≈ 4.5 [59] < 5.4 = pH), which led to both electrostatic repulsion among the COO^−^ groups and increase of osmotic pressure due to the formation of electrical charges inside the hydrogel [60]. This was the only phenomenon occurring in the case of H, HFS, and HHDK, and therefore, the SDe of silica-reinforced hydrogels was lower than that of the control hydrogel due to the crosslinking effect of inorganic particles (Figure 5).

Unlike FS and HDK, the other three reinforcing agents had a basic reaction in water (Table 1) due to the functional groups contained. As a result, HXLS, HXLG, and HMt contained COO^−^ groups from the synthesis step, which added to those formed at pH 5.4, leading to higher SDe as compared to H, HFS, and HHDK (Figure 5). The lower amount of COO^−^ groups of HMt (Figure 1) led to a smaller SDe than for HXLS and HXLG.

Further increase of the swelling medium pH to 7.4 determined a strong SDe increase in all cases, due to the formation of an increased number of COO^−^ groups within the hydrogels (Figure 5). It can be seen that, while the clay-reinforced hydrogels have maintained their advance in terms of SDe as compared to the control hydrogel for the reason discussed above, the SDe of silica-reinforced hydrogels displayed a dramatic increase at pH 7.4, exceeding the SDe of all the other hydrogels. This may be explained by their weakly acidic character (pKa ≈ 6–6.5 of the Si-OH groups on the surface of pyrogenic silica) [30], which meant they were not ionized at pH 5.4 and therefore they did not alter the water absorption of HFS and HHDK, but at the weak basic pH 7.4 they became ionized, which resulted in an increased water absorption.

The pH rise to 9.5 surprisingly led to a decrease in the SDe values for all hydrogels, while the hydrogel SDe order noticed at pH 7.4 was generally preserved (Figure 5). This phenomenon has been previously reported [61,62] in the case of PMAA hydrogels, but no explanation was provided. We do not have a clear explanation of this effect at this moment. As the FTIR spectra at pH 7.4 and 9.5 did not differ too much (Figure 1), we may only assume that an ionization degree reduction of the hydrogel COO^−^ groups may have happened, although the ionic strength of the swelling media was the same at these pH values.

### 2.3. Viscoelastic Properties

**Figure 6:**
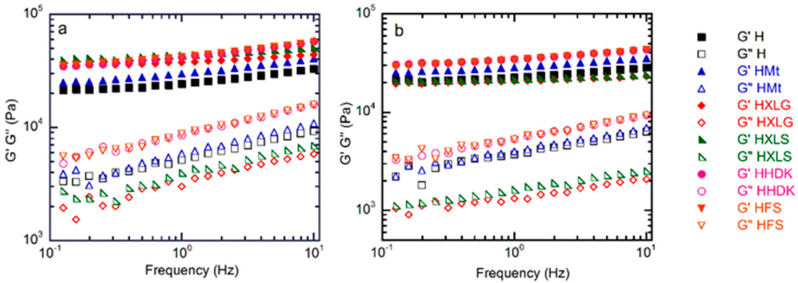
Frequency sweep rheological measurements on the (nano)composite hydrogels (**a**) in the as-prepared state and (**b**) after swelling at equilibrium.

The viscoelastic properties of the (nano)composite hydrogels were investigated, both in the as-prepared state, when the composition of the hydrogel was the same as in the precursor solution, and after equilibrium swelling in deionized water (pH 5.4). The frequency sweep measurements showed, in all cases, G’ higher than G” over the whole investigated range, thus confirming the crosslinked character of the hydrogels (Figure 6). In addition, G’ and G” increased with frequency, which is characteristic for networks with wide meshes [63].

**Figure 7:**
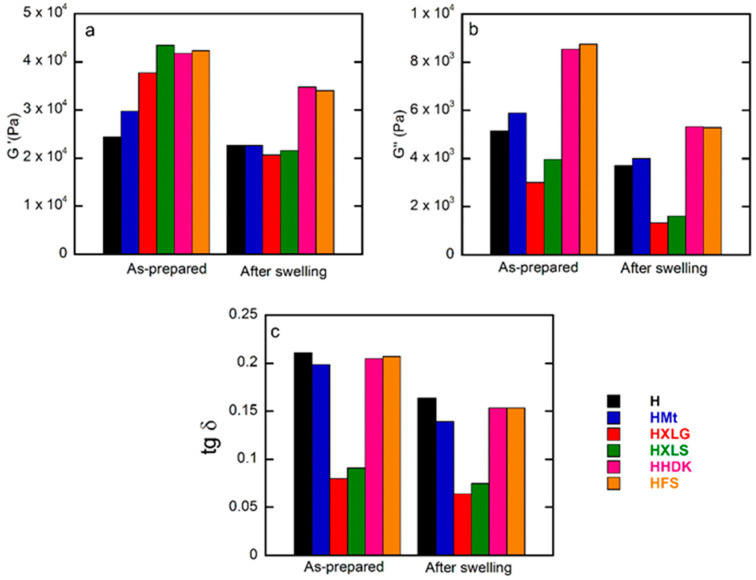
Frequency sweep rheological measurements on both as-prepared and equilibrium-swelled (pH = 5.4) hydrogels: (**a**) storage modulus; (**b**) loss modulus; (**c**) loss factor. Values obtained at 1 Hz.

The inclusion of reinforcing agents within the PMAA hydrogel matrix improved the mechanical properties, as confirmed by the rheological measurements on as-prepared hydrogels. The G’ values of these hydrogels (Figure 6a and Figure 7a) followed roughly the same order as the crosslinking density calculated for the swelled hydrogels at pH 1.2, thus confirming the previous results.

The same measurements on hydrogels swelled at equilibrium in DI (pH 5.4) showed, as expected, a decrease in G’ due to increased swelling (Figure 6b and Figure 7b) [64]. The G’ values obtained for the hydrogels reinforced with various fillers displayed an inverse tendency in relation to their swelling degree, i.e., the hydrogels with the lowest swelling degree (HFS, HHDK, Figure 5) showed the highest G’ (Figure 6a), while the hydrogels with the largest SDe (HXLG, HXLS, Figure 5) showed the lowest G’ values (Figure 7a).

The G’ and G” values at 1 Hz were used to calculate the loss factor (tan δ = G”/G’, Figure 7c). The tan δ values less than 1 obtained for all our hydrogels represented an additional confirmation of their crosslinked character. Additionally, tan δ greater than 0.15 in the case of H, HMt, HHDK, and HFS (Figure 7c), combined with the frequency dependency of G’ and G” (Figure 6), indicated their weak gel character [65], in both as-prepared and equilibrium-swollen (pH 5.4) states, although the G’ values were in the range of tens of kPa. Stronger hydrogels, with a more elastic character than the others, were obtained with XLG and XLS as the fillers, as indicated by the tan δ values less than 0.1 in both as-prepared and equilibrium-swollen states.

### 2.4. Absorption Properties of Cationic Dyes

Composite hydrogels have begun to gain more and more ground over time as potential candidates for use in the treatment of contaminated water [23,38]. Contamination with dyes from various fields, such as paper printing and textiles, plastics, and food and cosmetics industries is one of the biggest problems of environmental pollution [66]. Methylene blue (MB) and crystal violet (CV) are two of the most-used dyes in these industries. They belong to the category of cationic dyes containing the azo chromophore group and represent a concern for society due to their complex structure and non-biodegradable nature. They also hinder the penetration of sunlight into water, which affects living organisms [67].

**Figure 8:**
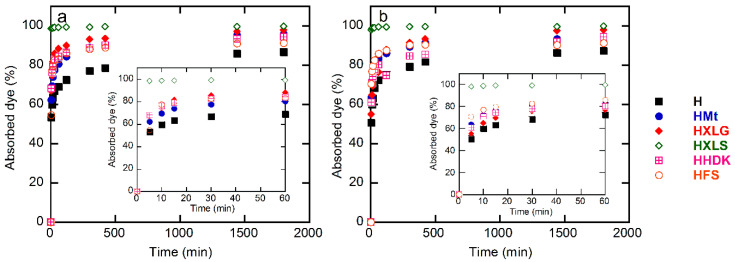
Time dependence of the percentage of dye absorbed by the (nano)composite hydrogels: (**a**) methylene blue; (**b**) crystal violet. Initial dye concentration = 20 mg/L; xerogel amount = 0.003 g; dye solution volume = 30 mL; temperature = 25 ± 0.5 °C.

**Figure 9:**
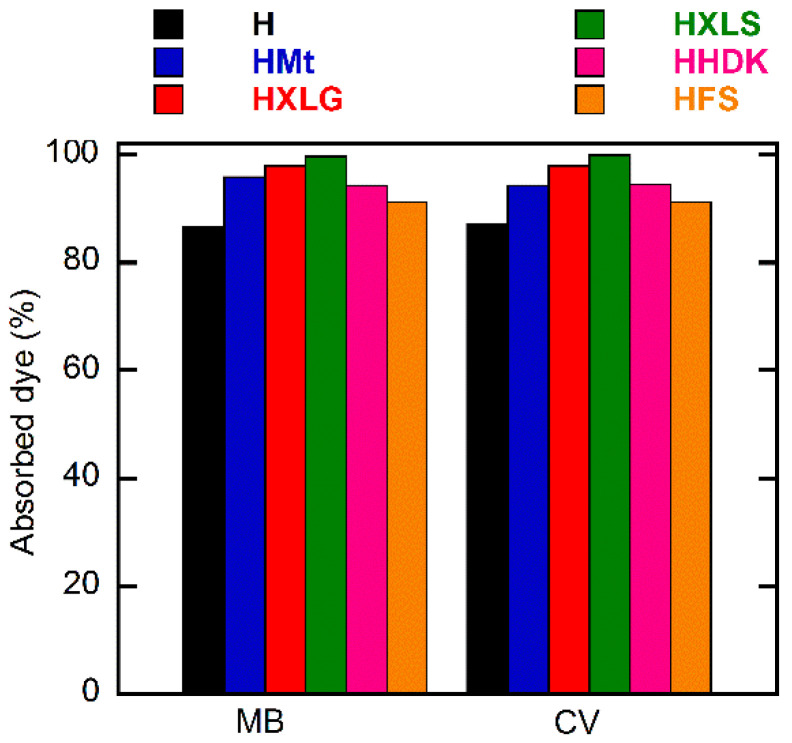
Dependence of the percentage of dye absorbed on reinforcing agent. Initial dye concentration = 20 mg/L; xerogel amount = 0.003 g; dye solution volume = 30 mL; temperature = 25 ± 0.5 °C; time = 30 h.

MB and CV were used within the present work as cationic dye models in aqueous solutions of 20 mg/L concentration in order to investigate the absorption properties of the synthesized composite hydrogels. The study of dye absorption by the (nano)composite hydrogels showed similar results in the case of both dyes, but the behavior of the hydrogels was different. Thus, HXLS showed the highest rate of dye absorption among all hydrogels, practically the entire amount of dye being absorbed from the solution in less than 30 min (Figure 8), a performance that was not achieved by the other hydrogels, even after 30 h (Figure 9).

For both dyes, a lowest absorption rate was displayed by the control hydrogel. This demonstrated the overall contribution of the reinforcing agent to the absorption process, through both its chemical structure and effect upon the hydrogel swelling degree.

In the case of PMAA hydrogels, the absorption of the cationic dyes was determined mainly by the carboxylate groups formed in the hydrogel in deionized water (pH 5.4), as explained earlier, which attract the positively-charged dye [68]. The control hydrogel, HFS, and HHDK contained only carboxylate groups from the ionization of MAA units in water, while hydrogels reinforced with clays (HMt, HXLG, HXLS) comprised additional carboxylate groups due to the clay basic reaction in water. In addition, the clay network also contained anionic groups that rapidly ionize in water, leading to the fast absorption of the cationic dye by an ion exchange process involving the positively-charged inorganic counterions of the clay [36]. Due to its preparation procedure, Laponite XLS also contains tetrasodium pyrophosphate, which represents additional anionic charges within the hydrogel, leading to an increased rate of dye absorption (Figure 8).

**Figure 10:**
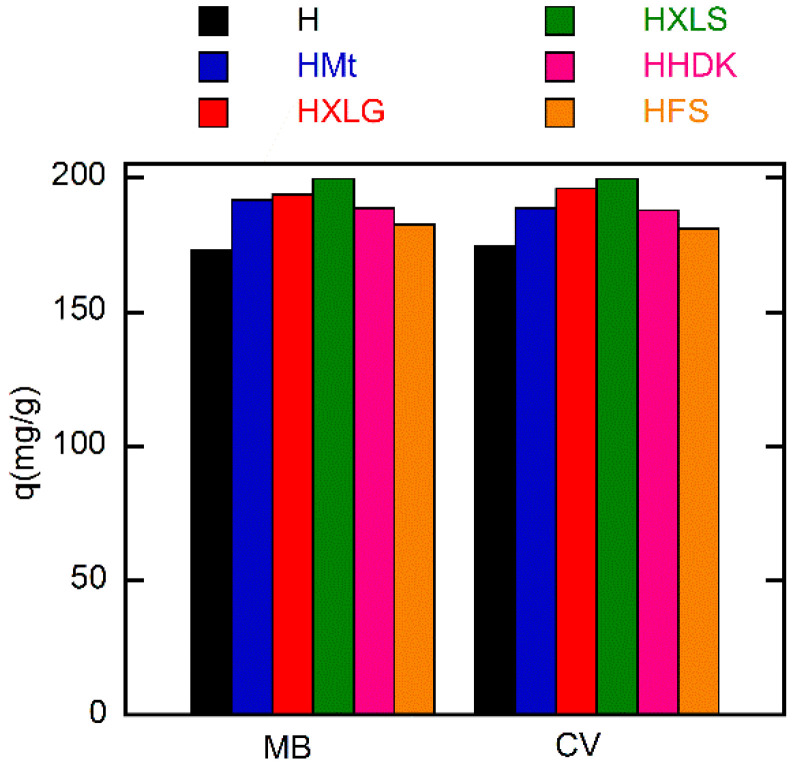
Dependence of the absorption capacity on the filler of the (nano)composite hydrogels. Initial dye concentration = 20 mg/L; xerogel amount = 0.003 g; dye solution volume = 30 mL; temperature = 25 ± 0.5 °C; time = 30 h.

Although FS and HDK do not influence the pH of deionized water (Table 1), the weakly acidic groups on the surface may contribute to the absorption of the dye. As a result, HFS and HHDK displayed a higher absorption rate as compared to the control hydrogel. The absorption curves showed in all cases a high absorption rate of the dye in the first 60 min, after which it considerably decreased (Figure 8). At the end of the investigated period (30 h), the absorption of the dye was over 85% in all cases, the lowest value being recorded for the control sample and the highest in the case of HXLS (Figure 9). The amount of dye ρ (mg) absorbed by 1 g of hydrogel after 30 h also depended on the nature of the reinforcing agent, decreasing for both dyes in the order HXLS > HXLG > HMt ≈ HHDK > HFS > H, from approximately 199.5 mg/g (MB)/196 mg/g (CV) in the case of HXLS to approximately 173 mg/g (MB)/174 mg/g (CV) for H (Figure 10). This represents a further confirmation of the beneficial contribution of the reinforcing agents described here to the absorption of cationic dyes.

## 3. Materials and Methods

### 3.1. Materials

Laponite XLG and Laponite XLS (BYK Additives & Instruments, kindly donated by Cosichem & Analytical, Romania), the two commercial brands of hydrophilic pyrogenic silica (fumed silica, FS, Sigma and HDK N20 pyrogenic silica, HDK, Wacker Silicones), methacrylic acid (MAA, Jansen Chemistry, 99%), N,N’-methylenebisacrylamide (BIS, 99%, Sigma Aldrich), ammonium persulfate (APS, Sigma Aldrich, 98%), methylene blue (MB, Loba Chemie, 95%), and crystal violet (CV, Loba Chemie, 96%) were used as received. Unmodified montmorillonite (Mt, Cloisite Na, a gift from Southern Clay Products Inc. Gonzales, TX, USA) was purified by the method previously described [43]. The characteristics of both reinforcing agents in water and their aqueous dispersions, determined by us, are displayed in Table 1. Deionized water (DI, 18.2 MΩ resistivity) was employed as the solvent for all syntheses. The swelling investigations were performed in DI (pH 5.4), HCl solution (pH 1.2), PBS solution (0.01 M, pH 7.4) obtained from tablets (Phosphate Buffered Saline, Sigma) and NaHCO_3_-Na_2_CO_3_ buffer (0.01 M, pH 9.5), whose ionic strength was adjusted by adding NaCl to make it equal to that of PBS (I = 0.17).

### 3.2. Synthesis of Hydrogels

The procedure employed was similar to the one previously described in reference [43]. The hydrogels were obtained by the radical copolymerization of MAA (15 wt% to the whole reaction mass) and BIS (2 mol% based on MAA) in the presence of APS (1 mol% based on MAA) as an initiator in aqueous solution. For the preparation of composite hydrogels, the calculated amount of reinforcing agent (1.5 wt% based on the whole hydrogel composition) was dispersed in DI for 24 h, and then MAA and BIS were added, followed by the initiator. The final reaction mixture was injected into a mold made up of two glass plates separated by a 1-mm thick Teflon gasket and glued with silicone. The mold was then placed in a thermostatted bath at 60 °C for 5 h. At the end of the polymerization time, the mold was removed from the thermostatted bath and allowed to cool to room temperature. Discs with diameters of 20 mm and 8 mm were cut from the material resulted after opening the mold. Part of the 20-mm discs were immediately subjected to rheological analysis, while the rest were analyzed after undergoing a purification-swelling process in DI for 7 days, together with the 8-mm discs, with a daily change of water.

To determine the monomer conversion, part of the hydrogel was first dried in atmosphere and then over anhydrous CaCl_2_ to a constant mass and weighed (W_o_). The dry xerogel was then immersed for purification in excess DI, which was changed daily for 7 days, dried again, then the final mass (W_ext_) was recorded. The monomer conversion (C%) was calculated according to Equation (1):

C% = (W_ext_ − W_AR_)/(W_o_ − W_AR_)∙100 (1)

where W_AR_ is the amount of reinforcing agent contained in the material employed to determine the conversion.

### 3.3. Crosslinking Density and Average Molecular Weight between Crosslinks

The average molecular weight between crosslinks (${\bar{M}}_{c}$) and the crosslinking density *(*$\rho_{c}$) were estimated in the case of hydrogels swelled at pH 1.2 in order to avoid the influence of the basic character of the filler aqueous dispersion on the swelling degree of the composite hydrogels. At pH 1.2, all the MAA units within the hydrogel were in acid form, and therefore the hydrogels can be considered as non-ionic. In the case of non-ionic hydrogels and neglecting the contribution of the chain-end defects, ${\bar{M}}_{c}$ can be calculated according to Equation (2) [64,69]:

(2) $${\bar{M}}_{c}=-\frac{\left(1-\frac{2}{f}\right)V_{1}\upsilon_{2r}^{2/3}\upsilon_{2m}^{1/3}}{\bar{\upsilon }\left[\ln\left(1-\upsilon_{2m}\right)+\upsilon_{2m}+{\chi \upsilon }_{2m}^{2}\right]}$$

where f is the functionality of the crosslinking site (f = 4), V_1_ represents the molar volume of the solvent (for water V_1_ = 18 cm^3^/mol), $\upsilon_{2r}$ and $\upsilon_{2m}$ represent the polymer volume fraction of the as-prepared (relaxed state) hydrogel and the hydrogel swelled at equilibrium, respectively, $\bar{\upsilon }$ is the specific volume of the polymer, calculated as 1/$\rho_{p}$ (cm^3^/g), while χ represents the Flory polymer–solvent interaction factor (0.48 for water—PMAA at 0% degree of ionization [70]). Both $\upsilon_{2r}$ and $\upsilon_{2m}$ were calculated by excluding the amount of filler contained by hydrogel and xerogel.

The polymer volume fractions ($\upsilon_{2r},\upsilon_{2m}$) were calculated using Equation (3) [71]:

(3) $$\nu_{2r/m}={\left[1+\frac{\rho_{p}}{\rho_{s}}\times \left(\frac{W_{h}}{W_{x}}-1\right)\right]}^{-1}$$

where $\rho_{p}\;$ and $\rho_{s}$ are the polymer and solvent densities (1.00 g/cm^3^ in the case of water), respectively, while W_h_ and W_x_ represent the mass of the hydrogel at equilibrium swelling ($\upsilon_{2m}$) or under as-prepared conditions ($\upsilon_{2r}$), and the mass of the corresponding xerogel, respectively, after excluding the filler in both cases. The polymer density was determined by the picnometer method, using Equation (4) and toluene as the non-solvent [72]. The polymer density represents the density of the H xerogel (corresponding to the hydrogel without filler).

(4) $$\rho_{X}=\frac{m_{X}\rho_{T}}{m_{a}+m_{X}-m_{b}}$$

where $m_{X}$ is the xerogel weight, $\rho_{T}$ is the density of toluene, determined by us with the picnometer, while $m_{a}$ and $m_{b}$ represent the weights of the picnometer filled with toluene and of the picnometer filled with toluene and containing the xerogel sample, respectively.

The crosslinking density of the hydrogels was calculated according to Equation (5) [71]:

(5) $$\rho_{c}=\frac{1}{\bar{\upsilon }\cdot {\bar{M}}_{c}}$$

### 3.4. Swelling Degree

To determine the swelling degree, pre-weighed xerogels (W_x_) were immersed in 40 mL of swelling solution and kept at 25 ± 0.5 °C for 72 h. At the end of the time interval, the hydrogels were removed from the solution, wiped superficially with filter paper, and weighed (W_H_). The equilibrium swelling degree (SDe) was calculated as the ratio of the amount of water absorbed to the mass of polymer in xerogel according to Equation (6):

SDe (g/g) = (W_H_ − W_X_)/(W_X_∙(100 − %A_R_)/100) (6)

where %A_R_ is the percentage of the reinforcing agent in xerogel, calculated based on the amount of monomers and filler employed in the polymerization and the overall monomer conversion. Each experiment was carried out in duplicate, and the average value ± the error was calculated for each point and reported.

### 3.5. Absorption Properties

To test the absorption properties of the synthesized materials, two cationic dyes, methylene blue (MB, maximum UV absorption at λ = 664 nm [73]) and crystal violet (CV, λ = 591 nm [74]), were used as models. The absorption capacity of hydrogels was determined using xerogel powders obtained by grinding in a ball mill, followed by drying over anhydrous CaCl_2_. A precisely weighed amount of approximately 0.003 g of xerogel was immersed in a centrifuge tube containing approximately 30 mL of a 20 mg/L aqueous dye solution and kept at 25 ± 0.5 °C with continuous orbital stirring on a heating–cooling dry plate (Torrey Pines Scientific Inc., USA) for 30 h. The pH of the dye solutions was 5.0 in the case of MB and 5.1 for crystal violet. The xerogel amount/dye solution volume ratio was kept constant in order to be able to compare the results obtained for the different xerogels used. Solution samples of 2 mL volume were withdrawn at predetermined time intervals, and the dye concentration was determined by UV-VIS spectrometry (UV-VIS Thermo instrument) by comparison with a calibration curve. To keep the solution volume constant, the volume withdrawn was reintroduced into the centrifuge tube after each analysis. The percentage of dye absorbed at various time intervals (PC_t_) and the amount of dye absorbed by 1 g of hydrogel (ρ) were determined using Equations (7) and (8):

(7) $${\mathrm{PC}}_{t}\;(\%)=\frac{\mathrm{Co}-\mathrm{Ct}}{\mathrm{Co}}\times 100$$

(8) $$\rho \;(\mathrm{mg}/g)=\frac{\mathrm{Co}-\mathrm{Ct}}{m}\times V$$

where Co and Ct (mg/L) represent the initial dye concentration and the concentration at time t, respectively, m = amount of xerogel used (g), and V = volume of solution used (L).

### 3.6. Characterizations

The viscoelastic properties of the hydrogels were investigated in both as-prepared and equilibrium-swollen state by means of a Kinexus Pro rheometer (Malvern Instruments, UK, software 1.60), equipped with a Peltier element for temperature control. The rheological measurements were performed at 25 °C using a geometry with 20-mm parallel plates with rough faces to avoid slippage. The applied normal force was 0.5 N. The amplitude sweep measurements were performed at 1 Hz constant frequency, and a strain within the linear viscoelasticity region was selected to be used in the frequency sweep measurements.

FT-IR analyses were run on a Tensor 37 Bruker (Woodstock, NY, USA) equipment with a resolution of 4 cm^−1^ and accumulation of 16 scans. The X-ray diffraction measurements were carried out in continuous mode, at room temperature and atmospheric pressure, on a Rigaku Ultima IV (Tokyo, Japan) instrument with CuKα radiation (λ = 1.5406 Å), operated at 40 kV and 30 mA. The data were collected over the 2θ range 1–50° and a scanning speed of 1°/min. Xerogel powders were used in both cases.

The thermogravimetric analyses (TGA) were performed on a TA Q5000 IR (TA Instruments) equipment under nitrogen atmosphere (40 mL/min), the samples being heated from room temperature to 700 °C at a rate of 10 °C/min.

SEM micrographs were obtained using an environmental scanning electron microscope (ESEM-FEI Quanta 200, Eindhoven, The Netherlands). The analyses were carried out under low vacuum at 30 kV acceleration voltage. The swollen samples were previously freeze-dried in an ALPHA 1-2 LDplus lyophilizer, Martin Christ, Germany. The morphology of the nanocomposite xerogels were obtained by transmission electron microscopy (TEM) by means of a TECNAI F20 G² TWIN Cryo-TEM (FEI, USA) equipment. The dried hydrogels were milled to a fine powder. Lacey formvar/carbon 200 mesh copper grids from Ted Pella, Inc., Redding, CA, USA, were gently contacted with each powder and thoroughly shaken afterwards to remove large powder particles. The samples were analyzed in bright field mode at an acceleration voltage of 120 kV.

The particle size of the reinforcing agents was measured by dynamic light scattering (DLS). The zeta potential of each aqueous dispersion of nanoparticles with a concentration of 1.5% was also measured. The measurements were performed by using a Nano ZS ZEN3600 Zetasizer instrument, Malvern Instruments (Malvern, UK). The pH of the aqueous dispersions of the reinforcing agents was measured with a pH-meter (Hanna Instruments), which was previously calibrated.

## 4. Conclusions

The present study showed that both the structure of hydrogels and some of their properties, such as viscoelastic, swelling, or absorbent/depolluting properties, can be adjusted to meet the requirements of a certain application by the correct choice of the reinforcing agent. To demonstrate this, the effect of five silicon-based fillers (Laponite XLS and XLG, Mt, and two commercial brands of pyrogenic silica), which differed mainly in particle size and pH of the aqueous dispersion, upon the PMAA crosslinked hydrogels was compared. Thus, XRD and TEM analyses showed that the two Lap clays and Mt led to exfoliated and respectively intercalated nanocomposites, while pyrogenic silica formed agglomerations of spherical nanoparticles within the hydrogel. The distribution of the nanoparticles within hydrogels was in agreement with the way the filler particles dispersed in water, i.e., isolated sheets in the case of XLG and XLS, stacked clay layers for Mt, and agglomerated spherical nanoparticles in the case of FS and HDK, as revealed by the DLS measurements. The structure of PMAA hydrogels was also affected by the reinforcing agent through the pH of its aqueous dispersion. Thus, Lap and Mt, displaying a basic reaction in water, determined the formation of carboxylate groups on the PMAA chains during the hydrogel synthesis stage. This led to an increase of the SDe of the nanocomposite hydrogels as compared to the one without filler at pH values larger than the pKa of the MAA monomer units.

The effect of the filler on SDe depended on both its nature and the pH of the swelling medium. At strong acidic pH (pH 1.2), a slight crosslinking effect of the filler was noticed, the most pronounced effect being displayed by pyrogenic silica. At pH 5.4, SDe significantly increased for all samples, the most pronounced increase occurring for the hydrogels synthesized in the presence of Laponite XLS and XLG. The crosslinking effect of pyrogenic silica particles was still evident at this pH. At basic pH, i.e., 7.4 and 9.5, the SDe of the pyrogenic silica-reinforced hydrogels surprisingly exceeded the SDe of all the other hydrogels. Also unexpectedly, the SDe at pH 9.5 was lower than at 7.4 for all hydrogels.

At approximately constant swelling degree, the filler addition improved th mechanical properties of the (nano)composite hydrogels, while after equilibrium swelling (pH 5.4), the viscoelastic moduli values depended on the filler. The rheological measurements also showed that the strongest hydrogels were obtained in the case of Lap as the reinforcing agent.

The (nano)composite hydrogels synthesized displayed a different ability to decontaminate cationic dye-containing waters as a function of the filler included, with both the highest absorption rate and absorption capacity being displayed by the Laponite XLS-reinforced hydrogel. Additionally, the presence of the filler within the hydrogel increased, in all cases, the amount of dye absorbed.

## Figures and Tables

**Table 1:** Characteristics of the investigated fillers and their aqueous dispersions ^1^.

Filler Code	Average Particle Size (nm)	Polydispersity Index	Zeta Potential (mV)	pH of Aqueous Dispersion
FS	367	0.417	−22.8	5.8
HDK	570	0.481	−17.5	5.9
Mt	724.5	0.535	−37.7	9.6
XLG	45.8	0.591	−41.9	9.6
XLS	38.2	0.521	−49.2	9.5

^1^ Concentration of the aqueous dispersion = 1.5 wt%; room temperature; dispersing time in deionized water = 24 h.

**Table 2:** TGA/DTG results obtained for the investigated composite xerogels.

Hydrogel	Weight Loss (%)			Residue at 700 °C (%)
	0–120 °C	120–300 °C	300–700 °C	
H	0.90	17.38	73.78	7.92
HMt	0.59	15.07	68.87	15.42
HXLG	0.40	16.32	63.90	19.36
HXLS	0.60	16.73	64.13	18.50
HHDK	0.62	15.51	69.18	14.65
HFS	0.66	15.65	68.59	15.08

**Table 3:** Dependence of the structural parameters ${\bar{\;M}}_{c}$ and $\rho_{c}$ of the composite hydrogels on the reinforcing agent at pH = 1.2.

Hydrogel	${\bar{M}}_{c}\;(\mathrm{Da})$	$\rho_{c}\times 10^{-4}\;(\mathrm{mol}/{\mathrm{cm}}^{3})$
H	643.0	20.0
HMt	539.7	23.8
HXLG	440.3	29.2
HXLS	423.3	30.4
HHDK	271.2	47.4
HFS	266.2	48.3

## Data Availability

The data presented in this study are available on request from the corresponding author.

## Supplementary Materials

The following supporting information can be downloaded at: https://www.mdpi.com/article/10.3390/ijms231810320/s1.
