# Supplementary material for: Adjusting Some Properties of Poly(methacrylic acid) (Nano)Composite Hydrogels by Means of Silicon-Containing Inorganic Fillers
Source: Int J Mol Sci. 2022 Sep 7;23(18):10320. doi: 10.3390/ijms231810320 (PMC9499409; doi:10.3390/ijms231810320)
Supplement: Supplementary file 1 [file ijms-23-10320-s001.zip › ijms-1881855-Supplementary.pdf]

Supplementary Data

Adjusting some properties of poly(methacrylic acid) (nano)composite hydrogels by means of silicon-containing inorganic fillers

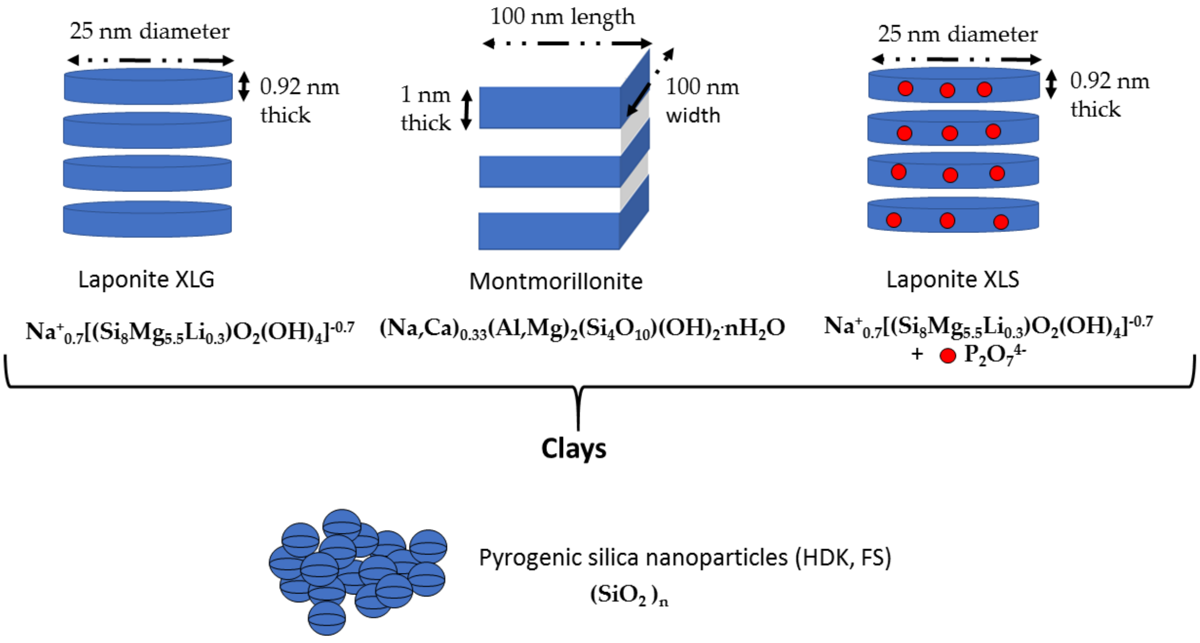

Figure S1. *Structure and chemical formula of the inorganic fillers used.*

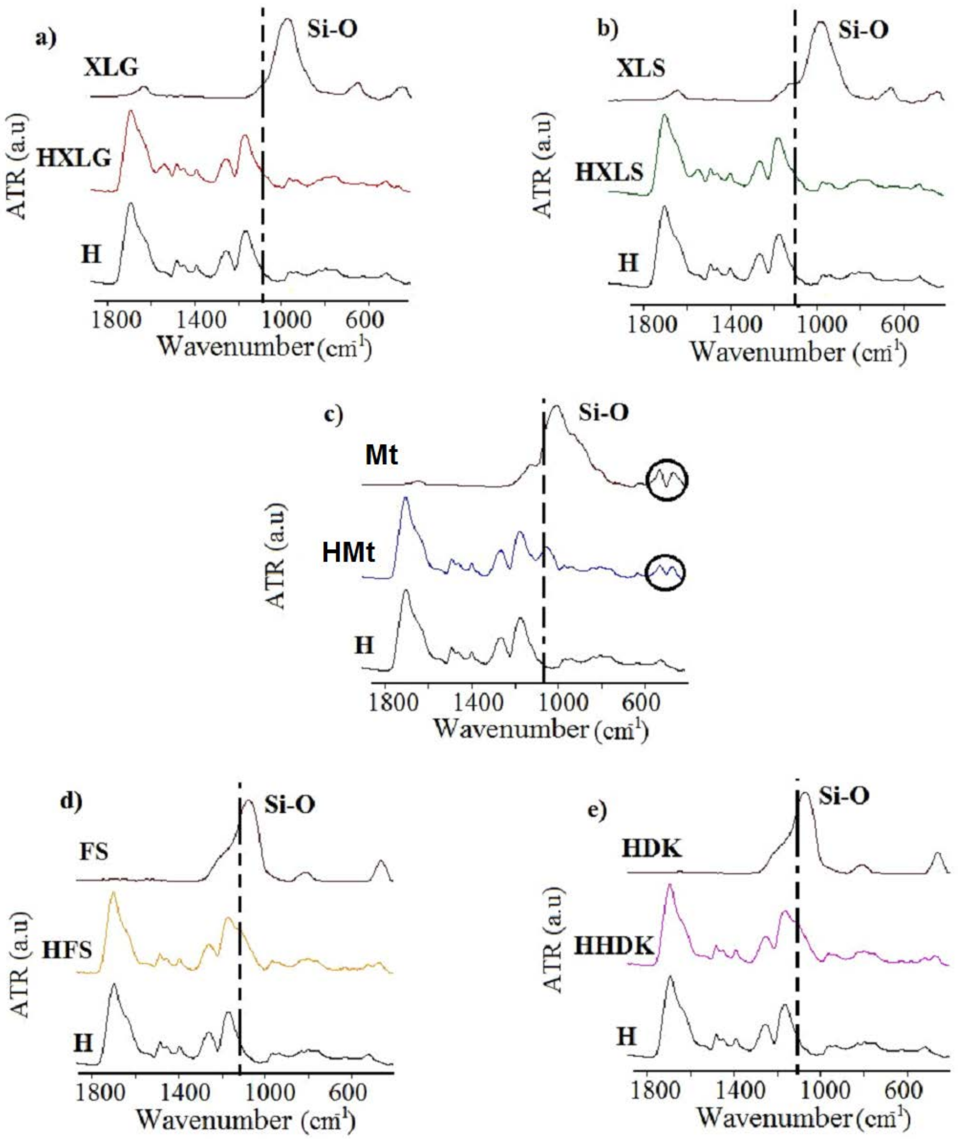

***Figure S2*. FT-IR spectra of the control hydrogel, composite hydrogels, and reinforcing agents used: a) XLG, b) XLS; c) Mt; d) FS; e) HDK. Hydrogels swelled at pH 5.4. XLS and XLG displayed the same characteristic bands at 977 cm^{-1} (Si-O), 647 cm^{-1} [Mg(Li)-O (OH)] and 1632 cm^{-1} (the OH bond from silanol groups)** [1]. *In the case of Mt, the peak characteristic of the Si-O-Si bond can be seen at 999 cm^{-1}, while for the silica nanoparticles (HDK and FS) a wide band at 1070 cm^{-1} characteristic to the Si-O-Si bond was visible. Shifts to higher wavenumbers of the Si-O peak in the FTIR spectra of xerogels values denoting solid-state interactions: XLG / XLS-977 cm^{-1} → HXLG / HXLS-1060 cm^{-1}, HDK / FS- 1070 cm^{-1} → HHDK / HFS-1110 cm^{-1}, Mt-999 cm^{-1} → HMt-1048 cm^{-1}.*

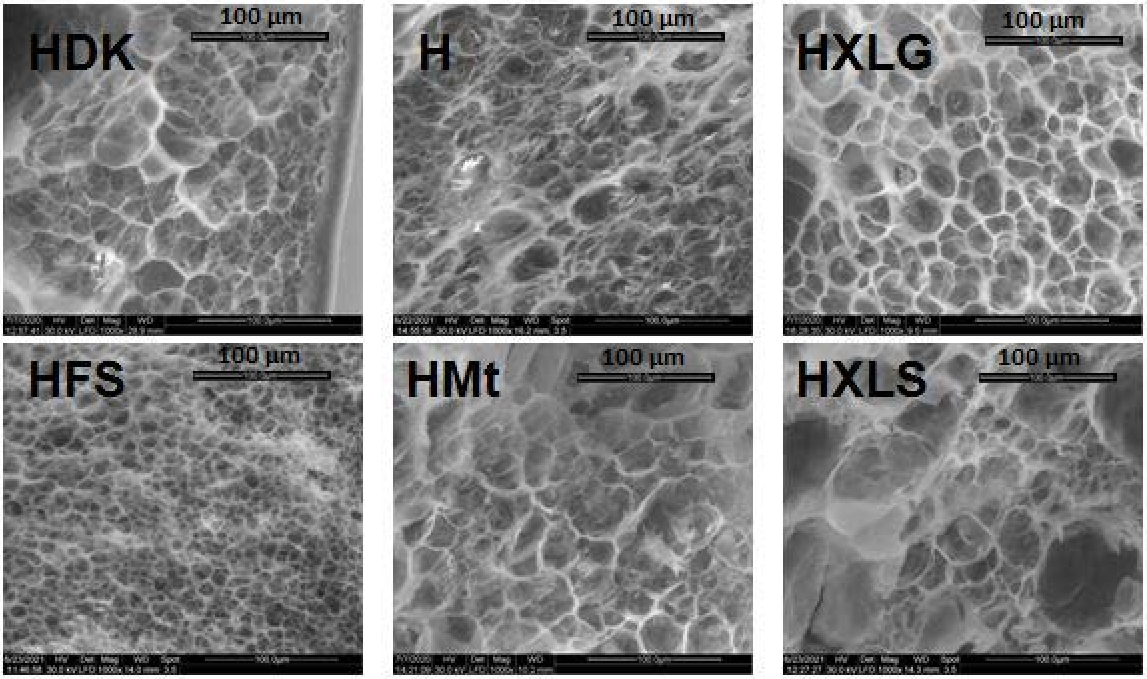

Figure S3. SEM images of lyophilized hydrogels (fracture surface).

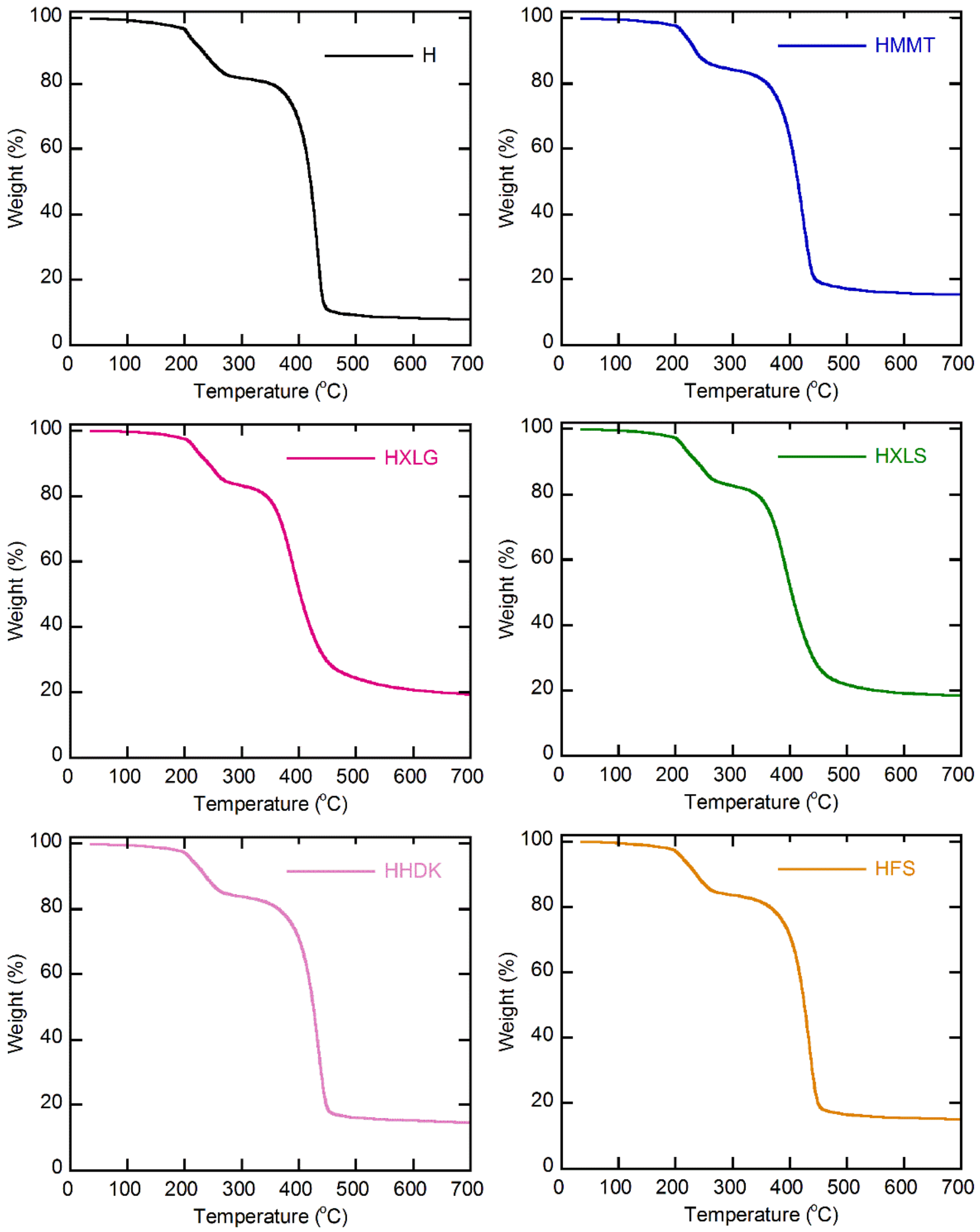

Figure S4. TGA curves for the composite hydrogels obtained.

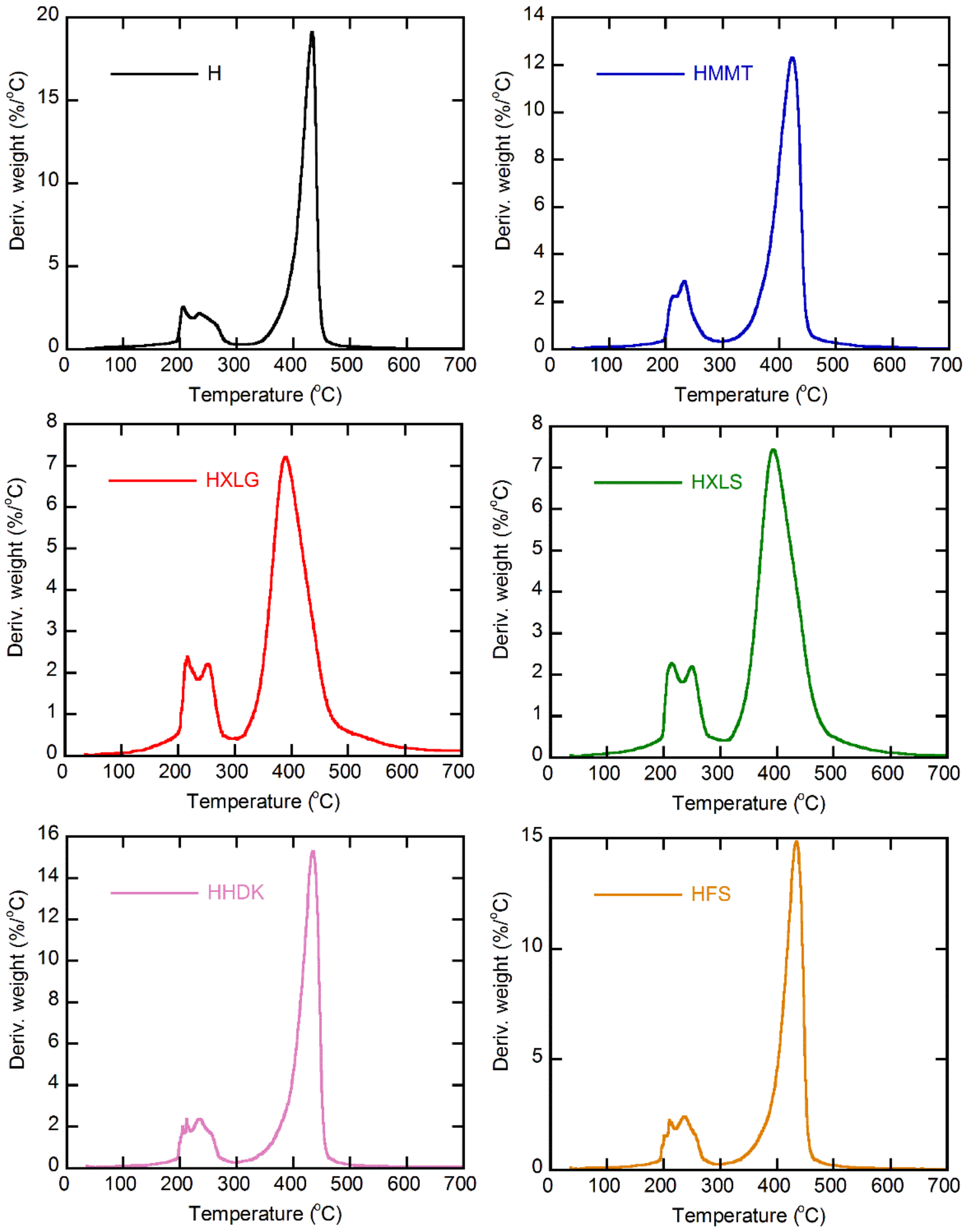

Figure S5. DTG curves for the composite hydrogels obtained.

References

1. Huang, H.; Liu, Z.; Yun, J.; Yang H.; Xu, Z. Preparation of Laponite Hydrogel in Different Shapes for Selective Dye Adsorption and Filtration Separation. *Appl. Clay Sci.* **2021**, *201*, 105936. https://doi.org/10.1016/j.clay.2020.105936
